# Standard values and relationship-specific validity of the Bielefeld Relationship Expectations Questionnaire (BFPE)

**DOI:** 10.1186/1471-2288-10-92

**Published:** 2010-10-13

**Authors:** Katja Petrowski, Hendrik Berth, Sören Paul, Gesine Grande, Yve Stöbel-Richter, Elmar Brähler

**Affiliations:** 1 Dresden University of Technology, Department of Psychotherapy and Psychosomatic Medicine, Fetscherstr. 74, D-01307 Dresden, Germany; 2 Dresden University of Technology, Department of Medical Psychology and Medical Sociology, Fetscherstr. 74, D-01307 Dresden, Germany; 3 HTWK Leipzig, Department of Social Welfare, PF 301166, D-04251 Leipzig, Germany; 4 University of Leipzig, Department of Medical Psychology and Medical Sociology, Phillip-Rosenthal-Str. 55, D-04103 Leipzig, Germany

## Abstract

**Background:**

The Bielefeld Partnership Expectations Questionnaire (BFPE) is a tool to assess attachment in the romantic relationships of adults. The attachment styles are operationalized as configuration patterns of scale scores. While convergent validity has already been investigated, discriminant validity is still lacking confirmation.

**Methods:**

The present sample (n = 1509) is representative for the German population aged 18 to 50. The mean age was 34.6 years. Most of the participants lived in a relationship (77.3 %). Discriminant validity was analyzed using a marital quality questionnaire (PFB), a social support questionnaire (F-Soz-U K-14), and a life satisfaction questionnaire (FLZ).

**Results:**

All the BFPE scales have a satisfying internal consistency between r = .79 and .86. Those individuals who showed a secure pattern, i.e. increased "Readiness for Self-Disclosure" and "Conscious Need for Care" as well as reduced "Fear of Rejection" experienced their partner as socially supportive, reported higher marital quality in all of its facets, and were more satisfied within the life-domains "family/children" and "relationship/sexuality". Standard values for each scale are presented.

**Conclusions:**

The BFPE has repeatedly been verified as a short, reliable, and valid instrument applicable to research practice with healthy individuals as well as within clinical contexts.

## Background

The attachment theory focuses on early interpersonal experience, affective patterns, and psychological development. In the attachment theory, John Bowlby [1] proposes that the early attachment experience with the primary caregiver is stored in an internal working model relied upon in attachment-relevant situations. This early attachment experience explains the development of normative relationship-specific behavior in later life. Hence, the attachment theory is widely used in research to explain normative as well as non-normative relationship-specific behavior.

Since the 1980's, more and more questionnaires have been developed to assess attachment in adults. The Bielefeld Relationship Expectations Questionnaire ('Bielefelder Fragebogen zur Partnerschaftserwartung', BFPE) [2,3], an additional German instrument for capturing attachment in romantic relationships, was developed for two reasons. First, with the BFPE, attachment can be operationalized dimensionally as well as categorically, hence allowing for the implementation of more statistical procedures and analyses. The Experience with Close Relationship Questionnaire (ECR) [4] has also seen the advantage of offering two different kinds of attachment data (dimensional and categorical). Second, most of the attachment questionnaires were exclusively developed for healthy samples, even though Bowlby's theory was used to describe non-normative as well as normative development. The BFPE and its items were developed for healthy as well as clinical samples, using a parallel version for the assessment of psychotherapy patients: the Bielefeld Client's Expectations Questionnaire ('Bielefelder Fragebogen zur Klientenerwartung', BFKE) [5].

The theoretical basis for the BFPE as well as the BFKE was the classic concept of attachment patterns by Mary Ainsworth [6]. She qualitatively defined patterns of attachment behavior and experience with attachment-specific situations. Herein, Ainsworth used observation protocols of mother-child-interactions during standardized separation and reunion. Based on the behavioral similarities among the children, they were grouped into attachment classifications. Höger and colleagues [2,3,5] used a similar approach. The questionnaire contains items asking for experience with attachment-relevant situations. The factor-analytically derived BFPE dimensional scales are "Conscious Need for Care", "Readiness for Self Disclosure", and "Fear of Rejection" showing moderate to large inter-scale correlations [5,7]. The single scale expressions of the BFPE were cluster-analytically grouped into five attachment patterns based on similarities in the sense of "prototypes". The five attachment clusters of the BFPE are called [3]: insecure-avoidant, partially secure, secure, (insecure) ambivalent-clinging and (insecure) ambivalent-withdrawing. They remain stable throughout different analytical methods (non-hierarchical k-means method and hierarchical method by Ward with a subsequent reclassification via discriminant analysis) and different samples [5,7].

Concerning convergent validity, the BFPE clusters were cross-validated using several attachment questionnaires based on different theoretical constructs. The German version of the Adult Attachment Scale (AAS) [8] is based on the vignettes of the Attachment Self-Report-Questionnaire [9]. This attachment classification was quite identical to the BFPE with a contingency coefficient of cc = .86 and κ = .82. The AAS dimension "Attachment Anxiety" is similar to the BFPE's "Fear of Rejection" whereas "Attachment Avoidance" is the scored-in-the-reverse version of "Readiness for Self-Disclosure" (see [3]). In addition, the German Attachment scale for Relationship (BinFR) [10] contains items from Simpson [11] as well as Brennan and Shaver [12]. Four of the five BFPE clusters were replicated whereas partially-secure attached individuals were classified as secure by the BinFR. Herein the correlation coefficients between the BinFR and the BFPE scales ranged between r = .49 and .82 (p < .001). Furthermore, the BFPE attachment patterns were correlated to recalled parental rearing behavior (FEE) [13] and differed significantly in the typical configurations of parental rearing behavior. As postulated by the attachment theory, secure attachment patterns are linked to positive experience with parental rearing while negative memories are linked to insecure attachment patterns and to more severe attachment-specific problems in relationships. Convergent validity can thus be supported.

A large body of literature examines the connections between attachment and relationship characteristics, e.g. relationship quality, social integration, and life satisfaction. A higher relationship quality is accompanied by a secure attachment pattern [14]. A lower relationship quality is associated with attachment avoidance (r = -.51) as well as attachment anxiety (r = -.21) [15]. The quality of the relationship in partnerships is closely connected to social support and adult life satisfaction. Attachment patterns predict how the individuals mobilize and use relationships [16]. Securely attached individuals perceive others as available, use more problem-focused strategies in conflicts, and are more satisfied with their current life. Insecure attachment, on the other hand, often occurs in negative relationships with more conflict, less support, and thus less life satisfaction [17]. Furthermore, the importance of attachment patterns increases with age [18]. Some possible explanations may be that attachment patterns mediate the influence of adverse childhood experiences on adult life satisfaction [17] or that attachment predicts perceived social support since a preoccupied pattern reduces the effect of support from partners [19]. It is therefore important to consider both life satisfaction and social support when interpreting attachment patterns in close relationships.

The present study investigates reliability, discriminant validity, and the standard values of the BFPE using a representative sample. Furthermore, the connections between attachment patterns and relationship quality, social support, and relationship-specific satisfaction, as published in the literature, are to be replicated for the dimensional BFPE scales. It must be assumed that securely-attached individuals, in contrast to insecurely-attached individuals, experience more support within their relationship. Since attachment is bonded to relationship satisfaction, which itself is associated with life satisfaction, it can be expected that securely-attached individuals are more satisfied with their life than insecurely-attached individuals.

## Methods

### Sample

The data descends from a population-representative German sample controlled for gender and education (*N *= 1509). It was collected in a multi-topic survey by the University of Leipzig. The participants were chosen by the random-route-method. They were visited at home and asked to answer several questionnaires. In the end, 59.6 % of all contenders completed the survey, 756 men (50.1 %) and 753 women (49.9 %) aged 18 to 50 (M = 34.6, SD = 9.02) participated, of whom 804 (53.3%) lived in the western part of Germany. Overall, 1104 individuals (77.3 %) lived in a relationship: 764 individuals (51.0 %) were married and lived together with their respective partner, 27 individuals (1.8 %) were married but lived apart, 132 individuals (8.8 %) were divorced, 19 (1.2 %) were widowed, and 557 (37.2 %) were single, i.e. did not have a partner. Further details regarding the sample have already been published [13].

### Instruments

The Bielefeld Relationship Expectations Questionnaire ('Bielefelder Fragebogen zur Parterschaftserwartung', BFPE) [2], a questionnaire to capture attachment patterns in relationships, was employed. The BFPE is the partnership-related parallel version of the Bielefeld Client Expectations Questionnaire (BFKE) [5]. The 30 items, and the warm-up item, are to be answered via a five-point rating scale ("not true at all" to "completely true"). A Varimax as well as an obliquely rotated principal component analysis yielded three factors that accounted for 48.2 % of variance and were affirmed by a subsequent confirmatory factor analysis with appropriate model fit [3]. The first factor called "Fear of Rejection" consists of items indicating a lack of self-esteem and the fear of being rejected, e.g. "Knowing myself as I do, I can hardly imagine that my partner will appreciate me" or "I sometimes think that my partner only loves me as much as I meet his expectations". The second factor, "Readiness for Self- Disclosure", is defined as a participant's ability and readiness for talking about inner feelings, e.g. "I prefer to talk with my partner about facts rather than about feelings" or "It's fairly easy for me to tell my partner about myself: my feelings, wishes, and needs". The third factor, "Conscious Need for Care", indicates a person's declared desire for the therapist's or partner's attention and care, e.g. "Being separated from my partner (e.g., traveling, business) makes me feel nervous and uncomfortable" or "It's important for me that my partner thinks of me often, even when we are not together". English versions of the BFKE and the BFPE can be found in Pollack, Wiegand-Grefe & Höger [3]. For the two scales "Fear of Rejection" and "Readiness for Self- Disclosure", Höger [2] reported good reliability (coefficient alpha .88 and .89, split-half reliability .91 an .89) and good mean corrected item-total correlations of .59 an .61, respectively. The "Conscious Need for Care" scale showed satisfactory results with coefficient alpha of .77, split-half reliability of .77 and a mean corrected item-total correlation of .47.

To assess relationship quality, the Marital Quality Questionnaire ('Partnerschaftsfragebogen'; PFB) by Hahlweg [20,21] was applied. This instrument consists of 30 items like "He/She reproaches me for mistakes I've done in the past" or "After going to bed, we cuddle up". They are rated with a four-point rating scale ("never/very seldom" to "very frequently") and can be grouped into the three ten-item scales "quarreling", "tenderness", and "togetherness/communication". An additional item with a six-point rating scale (from "very unhappy" to "very happy") is defined as the "global happiness estimation". The general score of relationship quality is gained by adding the scale values of tenderness and of communication [21]. All three scales showed good reliability for the individual scores (coefficient alpha .88 to .93) as well as for the main score (.95). The discriminant and predictive validity can be described as good. The original standardization sample consisted of N = 532 individuals, a new standardization followed in 2001 by Hinz and colleagues [22] with N = 1114 individuals.

The Social Support Questionnaire ('Fragebogen zur Sozialen Unterstützung, F-SozU [23-25]) contains statements about social contacts rated by a five-point Likert scale ("not true at all" to "completely true"). In the present study, a short form with 14 items was used, which had formerly been developed from the 54-item-standard version by using item- and factor-analytical methods during a representative study (F-SozU K-14) [26,27]. The statements refer to the domains of emotional support (e.g. "Whenever I am really depressed, I know who to go to."), practical support (e.g. "Whenever I am really stressed, someone takes tasks off my shoulders."), and to social integration (e.g. "There is a community of people- circle of friends, clique - to which I feel affiliated"). Psychometric properties were frequently investigated (e.g. [23,24,26]). The scale value, as the complete score of the social support experienced, is calculated as the mean rating for all scale items. In this way, the comparability with enlarged F-SozU-versions is supported. Standard values from representative samples are available for all questionnaire versions.

The Life Satisfaction Questionnaire ('Fragebogen zur Lebenszufriedenheit'; FLZ) [28] consists of modules for each, general life satisfaction and health, however, only the two relationship-relevant dimensions of general life satisfaction (family life/children and partner relationship/sexuality) are analyzed [29]. All respondents rate the subjective importance of each dimension and their present satisfaction with each dimension on scales from 1 (not important/dissatisfied) to 5 (extremely important/very satisfied). The internal consistency (coefficient alpha) of the dimensions varies between .82 and .95 [28]. The factor analysis and the relation of the dimensions to several personality traits corroborate validity. Present stanine standard values are based on a population-representative survey of N = 2870 individuals aged 14 to 92.

All the participants volunteered and received a data protection declaration in agreement with the Helsinki Declaration. The study was approved according to the ethical guidelines of the "German Professional Institutions for Social Research" [Arbeitskreis Deutscher Markt- und Sozialforschungsinstitute, Arbeitsgemeinschaft Sozialwissenschaftlicher Institute, Berufsverband Deutscher Markt- und Sozialforscher]. Therefore, obtaining additional ethical approval was not necessary.

## Results

### Reliability and corrected item-total correlations

Table 1 shows mean values, standard deviations, reliabilities, and corrected item-total correlations, i.e. the correlation of an item to the scale-wise sum of item scores without including the item in question. Scoring the items between 1 and 5 as applicable, on average, the participants reported low to moderate "Fear of Rejection", moderate "Readiness for Self Disclosure" and moderate to high "Conscious Need for Care".

**Table 1:** Reliabilities and item-total correlations of BFPE scales

					Reliabilities			Corrected ITCs	
					
Scale	Items	***N***	***M***	***SD***	Split Half	Coefficient α	minimal	maximal	mean
Fear of Rejection	11	1506	24.0	7.55	.85	.86	.40	.64	.52
Readiness for Self Disclosure	11	1497	27.6	6.74	.80	.79	.27	.53	.40
Conscious Need for Care	8	1511	25.4	5.77	.77	.79	.42	.58	.50

Note: Item scoring range is 1 to 5; split-half reliabilities adjusted after Spearman-Brown; ITCs = item-total correlations

Split-half reliability and internal consistency (coefficient alpha) were good for "Fear of Rejection". For "Readiness for Self Disclosure" and "Conscious Need for Care", they were good to satisfactory.

Whether an item assesses the same as its theoretically associated scale is measured via corrected item-total correlations. Each item was acceptably correlated to its respective scale with r around .50, while item 6 (r = .34) and item 23 (r = .27) were least correlated to their respective scale, "Readiness for Self Disclosure".

### BFPE - intercorrelations and relationship-specific validity

Pearson product moment correlation coefficient between the BFPE scales and the likewise used questionnaires PFB, F-SozU (K-14), and FLZ are presented in Table 2.

**Table 2:** Pearson product moment correlation coefficients between BFPE and PFB, F-SozU (K-14), FLZ

Scales	Fear of Rejection	Readiness for Self Disclosure	Conscious Need for Care
BFPE			
Readiness for Self Disclosure	-.63**	-	-
Conscious Need for Care	.16**	.15**	-
PFB			
Quarreling	.51***	-.38***	.06*
Tenderness	-.22***	.42***	.30***
Togetherness/communication	-.31***	.47***	.32***
Global happiness	-.43***	.54***	.29***
F-SozU (K-14)			
Mean complete score	-.32***	.43***	.20***
FLZ			
Importance of family/children	-.19***	.30***	.24***
Importance of partnership/sexuality	-.14***	.31***	.27***
Satisfaction with family/children	-.31***	.33***	.12***
Satisfaction with partnership/sexuality	-.30***	.39***	.15***

Note: * *p *< .05; ** *p *< .01; *** *p *< .001 (two-sided); *n *= 1372 to 1529; [.1 <*r *< .3 = small correlation, .3 <*r *< .5 = mediocre correlation, *r *> .5 = high correlation]

Generally speaking, a higher "Fear of Rejection" is accompanied by a much lower "Readiness for Self Disclosure". The remaining two correlations among BFPE scales were significant and positive, but of small magnitude.

The correlations between dimensions of marital quality from the PFB and the attachment dimensions were mostly in the moderate to large range. Low levels of "Fear of Rejection" and high levels of "Readiness for Self Disclosure" were associated with low levels of perceived partner "Quarreling" and thus with high levels of cooperativeness in seeking solutions to conflicts. Further, physical contact, shared activities, and the communication of positive affect within the relationship were moderately inversely related to "Fear of Rejection", and moderately positively related to both "Readiness for Self Disclosure" and "Conscious Need for Care". General Relationship quality was negatively related to "Fear of Rejection" and, therefore, positively related to "Readiness for Self Disclosure" and "Conscious Need for Care". Individuals who experienced their partner as socially supportive, as measured by the F-SozU (K-14), showed greater ability and willingness to speak about themselves and their needs than did persons with less supportive partners. The correlations of social support with "Fear of Rejection" and with "Conscious Need for Care" tended to be of smaller magnitude, although significant and in the predicted direction.

Individuals who experienced their partner as socially supportive, as measured by the F-SozU (K-14), showed an increased ability and willingness to speak about themselves and their needs. In contrast, only a mediocre correlation was found between social support and "Fear of Rejection" or "Conscious Need for Care".

The Life Satisfaction Questionnaire (FLZ) scales were, in general, moderately and positively intercorrelated, ranging from r = .06 (p = .03) to r = .66 (p = .00). In regard to the BFPE scales, it appears that the more individuals stated their satisfaction with family/children and relationship/sexuality, the less they showed "Fear of Rejection" and the more they showed "Readiness for Self Disclosure". Increased "Readiness for Self Disclosure" was also found in individuals who considered these domains important. "Conscious Need for Care", however, was only slightly connected to life satisfaction.

### Standard percentages

Table 3 contains computed sum scores, z-values, T-values and percentages for all the BFPE scales.

**Table 3:** Sum scores, z-values, T-values and percentages for every BFPE scale

Sum score	Fear of Rejection (*n *= 1506)			Readiness for Self Disclosure (*n *= 1497)			Conscious Need for Care (*n *= 1511)		
	*z*	*T*	PR	*z*	*T*	PR	*z*	*T*	PR
**8**	--	--	--	--	--	--	-3.01	20	0
**9**	--	--	--	--	--	--	-2.84	22	0
**10**	--	--	--	--	--	--	-2.67	23	1
**11**	-1.72	33	1	-4.04	10	0	-2.49	25	1
**12**	-1.59	34	3	-3.91	11	0	-2.32	27	1
**13**	-1.45	35	6	-3.77	12	0	-2.15	29	2
**14**	-1.32	37	9	-3.63	14	0	-1.97	30	3
**15**	-1.19	38	12	-3.49	15	0	-1.80	32	4
**16**	***-1.06***	***39***	***16***	-3.35	16	0	-1.62	34	5
**17**	***-0.92***	***41***	***20***	-3.22	18	0	-1.45	35	8
**18**	***-0.79***	***42***	***24***	-3.08	19	0	-1.28	37	10
**19**	***-0.66***	***43***	***28***	-2.94	21	0	-1.10	39	14
**20**	***-0.53***	***45***	***33***	-2.80	22	0	***-0.93***	***41***	***18***
**21**	***-0.39***	***46***	***38***	-2.66	23	0	***-0.76***	***42***	***22***
**22**	***-0.26***	***47***	***44***	-2.52	25	1	***-0.58***	***44***	***27***
**23**	***-0.13***	***49***	***49***	-2.39	26	1	***-0.41***	***46***	***33***
**24**	***0.00***	***50***	***53***	-2.25	28	1	***-0.24***	***48***	***41***
**25**	***0.14***	***51***	***58***	-2.11	29	1	***-0.06***	***49***	***48***
**26**	***0.27***	***53***	***63***	-1.97	30	2	***0.11***	***51***	***55***
**27**	***0.40***	***54***	***67***	-1.83	32	3	***0.28***	***53***	***61***
**28**	***0.53***	***55***	***71***	-1.70	33	4	***0.46***	***55***	***67***
**29**	***0.67***	***57***	***74***	-1.56	34	6	***0.63***	***56***	***72***
**30**	***0.80***	***58***	***78***	-1.42	36	8	***0.80***	***58***	***78***
**31**	***0.93***	***59***	***81***	-1.28	37	10	***0.98***	***60***	***83***
**32**	***1.06***	***61***	***84***	-1.14	39	13	1.15	61	87
**33**	1.20	62	87	***-1.01***	***40***	***16***	1.32	63	91
**34**	1.33	63	91	***-0.87***	***41***	***21***	1.50	65	93
**35**	1.46	65	92	***-0.73***	***43***	***25***	1.67	67	95
**36**	1.59	66	94	***-0.59***	***44***	***30***	1.84	68	97
**37**	1.73	67	95	***-0.45***	***45***	***35***	2.02	70	98
**38**	1.86	69	96	***-0.32***	***47***	***39***	2.19	72	99
**39**	1.99	70	97	***-0.18***	***48***	***45***	2.36	74	100
**40**	2.12	71	97	***-0.04***	***50***	***49***	2.54	75	100
**41**	2.26	73	98	***0.10***	***51***	***53***			
**42**	2.39	74	98	***0.24***	***52***	***58***			
**43**	2.52	75	99	***0.38***	***54***	***62***			
**44**	2.65	77	99	***0.51***	***55***	***67***			
**45**	2.79	78	99	***0.65***	***57***	***72***			
**46**	2.92	79	100	***0.79***	***58***	***76***			
**47**	3.05	81	100	***0.93***	***59***	***80***			
**48**	3.18	82	100	***1.07***	***61***	***83***			
**49**	3.32	83	100	1.20	62	86			
**50**	3.45	84	100	1.34	63	90			
**51**	3.58	86	100	1.48	65	92			
**52**	3.71	87	100	1.62	66	95			
**53**	3.85	88	100	1.76	68	97			
**54**	3.98	90	100	1.89	69	98			
**55**	4.11	91	100	2.03	70	99			

Note: Area of normality (between PR 15.8 and PR 84.2; i.e. M +/- SD) is printed in bold italics.

## Discussion

On the one hand, the present study examined the psychometric properties of the BFPE with reference to a representative sample and, on the other hand, the evidence for its discriminant validity. Hence, the relationship between the BFPE attachment scales and relationship quality, social support within the relationship, and general life satisfaction were analyzed.

Comparing the psychometric properties of the BFPE to published studies, the following striking analogies and differences appear: The BFPE scales show low inter-correlations except for the highly negative correlation between "Fear of Rejection" and "Readiness for Self Disclosure". This association was also found in a healthy sample [2] and in a clinical sample using the BFKE [5]. However, Brennan, Shaver, and colleagues [4,12] reported zero correlations for their respective scales. Therefore, a detailed analysis of the underlying concepts should investigate these divergent results further. The correlation between "Fear of Rejection" and "Conscious Need for Care" is reported as two times higher in the healthy sample and as four times higher than in the clinical sample using the BFKE. The correlation between "Readiness for Self Disclosure" and "Conscious Need for Care" is minimal but positive in the healthy sample; in the clinical sample it is moderate but negative. The differences between the healthy and the clinical samples might have been due to different attachment distributions.

The good split-half reliabilities, internal consistencies, and the satisfactory mean corrected item-total correlations are nearly identical to the ones reported by Höger and Buschkämper [2] for "Fear of Rejection" (r = .91, α = .88, mean r_corrected ITC _= .59) and "Conscious Need for Care" (r = .76, α = .77, mean r_corrected ITC _= .47). They are still satisfactory, yet slightly lower for "Readiness for Self Disclosure" (r = .89, α = .89, mean r_corrected ITC _= .61).

Previous studies found evidence of convergent validity (see [2,3]) as they reported a huge content overlap with the Adult Attachment Scale [8] and Bartholomew's four-category model (see [3]) as well as high correlations with recalled parental behavior (FEE) [13].

This present study provides evidence for discriminant validity as the BFPE scales are moderately correlated to theoretically linked, distinct concepts, i.e. relationship quality, social integration, and life satisfaction. It was possible to replicate these connections. Therefore, these findings confirm the known effect that attachment patterns and the underlying dimensions influence life satisfaction, especially within close relationships, and mediate how social support within these relationships is perceived [17,19]. Hinne, Sanderman, and Sprangers [17] concluded that attachment patterns even mediate the influence of childhood recollections on life satisfaction. Thus, both the attachment theory and the empirical data underline the importance of the relation, which was examined in the present study. Moreira and colleagues [19] found evidence that attachment styles determine the perception of social support and moderate the impact of social support. Hence, the present correlations will be interpreted in reference to these frameworks.

With respect to general life satisfaction, the satisfaction in the realms family/children and relationship/sexuality is connected to a low "Fear of Rejection" and high "Readiness for Self Disclosure" whereas "Conscious Need for Care" does not show any meaningful relation to it.

Higher levels of perceived social support, i.e., experiencing the partner as less prone to prolonging conflict, is associated with lower levels of "Fear of Rejection" and higher levels of "Readiness for Self Disclosure" towards their partner. This mirrors previous findings by showing that secure attachment patterns are accompanied by positive and effective conflict-solving strategies [30,31].

Heightened "Readiness for Self Disclosure" and "Conscious Need for Care" are associated with marital quality, i.e., more physical contact, more shared activities, and increased communication. This underlines findings indicating a connection between a secure attachment pattern and high communicative quality [32,33] as well as a higher relationship quality in securely attached individuals [14]. One might hypothesize that the more individuals in this representative sample spoke about themselves and their own needs, the more they experienced their partner as socially supportive. It must be noted that "Fear of Rejection" showed an opposite pattern of correlations with relationship quality scales than "Readiness for Self Disclosure" did, i.e., it correlated strongly positive with "Quarreling" but negative with the remaining three scores. This shows the opposite nature of the two scales as being inter-correlated strongly negative while the small correlations between them and "Conscious Need for Care" show that the latter scale is probably independent.

In sum, the scale pattern with the most positive effects on all three constructs is the secure pattern, i.e., increased "Readiness for Self Disclosure", "Conscious Need for Care", and reduced "Fear of Rejection".

One limitation of this study is that the presented standard values of the instrument did not focus on any connection to patients with mental disorders nor did it report any details regarding the psychiatric symptoms in the present sample. This issue, however, needs to be addressed since it may be more than likely that the attachment patterns of individuals suffering from mental disorders have become altered [5,34]. To test this hypothesis and to account for possible alterations, future studies should concentrate on clinical samples as well as the assessment of psychiatric symptoms and applicable, present disorder-specific standard values. This step is indispensable for broad clinical application.

A more methodological limitation is "sentiment override" as the individuals asked about their partner and marriage tended to respond to the questionnaires more in terms of their sentiments than in terms of the items' manifest content [35]. As Pollack and colleagues pointed out, filling out an attachment questionnaire, i.e., directly being called on to remember partnership experience activates the inner working model and unconsciously regulates the access to this information [3]. The BFPE indeed assesses partnership-specific attachment by covering these response tendencies.

## Conclusions

In sum, the BFPE is a questionnaire that assesses adult attachment by explicitly asking for experiences from attachment-specific situations. This allows for attachment dimensions and attachment patterns to be analyzed. For the application in psychotherapy settings, the BFKE, a specific parallel version of the BFPE, is available, too. Repeated implementations of the BFPE are possible within broader clinical contexts. Lastly, as the representative data demonstrate, the BFPE is a reliable and valid instrument for measuring attachment patterns in adults.

## Competing interests

The authors declare that they have no competing interests.

## Authors' contributions

KP did the first and final draft of the manuscript and critically revised it for its intellectual content. HB, SP, GG, and YS substantially contributed to the analysis and the interpretation of the data. EB was responsible for the collection of the data and the general supervision of the research group. He substantially contributed to the conception and the design of the study as well as the acquisition of the data. All authors read and approved the final manuscript.

## Pre-publication history

The pre-publication history for this paper can be accessed here:

http://www.biomedcentral.com/1471-2288/10/92/prepub
